# The phytotoxin coronatine is a multifunctional component of the virulence armament of *Pseudomonas syringae*

**DOI:** 10.1007/s00425-014-2151-x

**Published:** 2014-08-26

**Authors:** Xueqing Geng, Lin Jin, Mikiko Shimada, Min Gab Kim, David Mackey

**Affiliations:** 1 Department of Horticulture and Crop Science, Ohio State University, Columbus, OH 43210 USA; 2 Department of Molecular Genetics, Ohio State University, Columbus, OH 43210 USA; 3 College of Pharmacy, Research Institute of Pharmaceutical Science, PMBBRC Gyeongsang National University, Jinju daero, Jinju, 660-751 Republic of Korea; 4 School of Agriculture and Biology, Shanghai Jiao Tong University, Shanghai, 200240 People’s Republic of China

**Keywords:** Phytotoxin, Coronatine, Plant hormones, Hormone crosstalk, Plant defense, Type III effectors

## Abstract

Plant pathogens deploy an array of virulence factors to suppress host defense and promote pathogenicity. Numerous strains of *Pseudomonas syringae* produce the phytotoxin coronatine (COR). A major aspect of COR function is its ability to mimic a bioactive jasmonic acid (JA) conjugate and thus target the JA-receptor COR-insensitive 1 (COI1). Biological activities of COR include stimulation of JA-signaling and consequent suppression of SA-dependent defense through antagonistic crosstalk, antagonism of stomatal closure to allow bacterial entry into the interior of plant leaves, contribution to chlorotic symptoms in infected plants, and suppression of plant cell wall defense through perturbation of secondary metabolism. Here, we review the virulence function of COR, including updates on these established activities as well as more recent findings revealing COI1-independent activity of COR and shedding light on cooperative or redundant defense suppression between COR and type III effector proteins.

## Introduction

Phytotoxins are microbe-produced secondary metabolites that interfere with and sometimes kill plant cells. They are either directly active or are produced as prototoxins that become activated by plant enzymes (Duke and Dayan 2011; Pruess et al. 1973; Uchytil and Durbin 1980). *Pseudomonas syringae* pathovars produce a repertoire of virulence effectors that are active inside plant cells, including numerous phytotoxins (Hogenhout et al. 2009). One class of effects mediated by phytotoxins is disruption of amino acid metabolism. For example, phaseolotoxin blocks the production of arginine by inhibiting ornithine transcarboxylase (Ferguson and Johnston 1980). Tabtoxin gets converted *in planta* to a glutamate analog that inhibits glutamine synthetase thus causing a buildup of ammonia and glutamine deficiency (Turner 1981; Uchytil and Durbin 1980). Other effects of phytotoxins are quite diverse and include perturbation of metabolism of lipids, sugars, and cell walls, synthesis of proteins and nucleic acids, membrane integrity and mitosis (Duke and Dayan 2011; Ferguson and Johnston 1980; Goudet et al. 1999; Pruess et al. 1973; Hoffman 1995; King and Calhoun 2009; Strobel et al. 1996; Thuleau et al. 1988; Walton 2006; Daub et al. 2005; Tanaka 1996). Another effect of phytotoxins is perturbation of hormone signaling. Phytohormones play key roles in a variety of physiologic and cellular processes, including numerous processes related to plant defense that have been extensively reviewed elsewhere (Bari and Jones 2009; Howe and Jander 2008; Katagiri and Tsuda 2010; Pieterse et al. 2009). While some phytotoxins likely perturb hormone signaling indirectly, COR directly engages JA-signal transduction proteins to co-opt hormone signaling.

COR is a polyketide phytotoxin produced by pathovars of *P. syringae*, including *alisalensis, atropurpurea, glycinea, maculicola, morsprunorum, porri*, and tomato (Bender et al. 1999; Gross and Loper 2009; Mitchell 1982; Mitchell et al. 1983; Preston 2000; Ullrich et al. 1993; Wiebe and Campbell 1993; Zhao et al. 2000; Cintas 2002) (for these and other bacterial strains discussed, refer to Table 1). Additionally, COR-analogs are produced by *Xanthomonas campestris* pv. *phormiicolai* (Tamura et al. 1992; Mitchell 1991). Consistent with infection of a diverse set of host plants by these pathovars and species, COR is a non-host specific toxin that causes diffuse chlorosis in a wide variety of plant species (Rohde et al. 1998; Brooks et al. 2004).

**Table 1:** Strains discussed in this review

Strain name	COR production	References
*P. syringae* pv. tomato	Yes	Bender et al. (1999), Cintas et al. (2002), Gross and Loper (2009), Mitchell (1982), Mitchell et al. (1983), Ullrich et al. (1993), Wiebe and Campbell (1993), Zhao et al. (2000)
*P. syringae* pv. *alisalensis*	Yes	
*P. syringae* pv. *atropurpurea*	Yes	
*P. syringae* pv. *glycinea*	Yes	
*P. syringae* pv. *maculicola*	Yes	
*P. syringae* pv. *morsprunorum*	Yes	
*P. syringae* pv. *porri*	Yes	
*Xanthomonas campestris* pv. *phormiicolai*	COR-analogs	Mitchell (1991), Tamura et al. (1992)
*P. syringae* pv. tomato (*Pto*) DC3000	Yes	Buell et al. (2003), Preston (2000)
*Pto* DC3000 mutant strains		
*Pto* cor-	No	Brooks et al. (2004)
*Pto*∆CEL	Yes	Alfano et al. (2000)
*Pto*∆CEL cor-	No	Geng et al. (2012)
*Pto* cor- *hrpS*	No	Thilmony et al. (2006)

## COR biosynthesis and structure

**Fig. 1:**
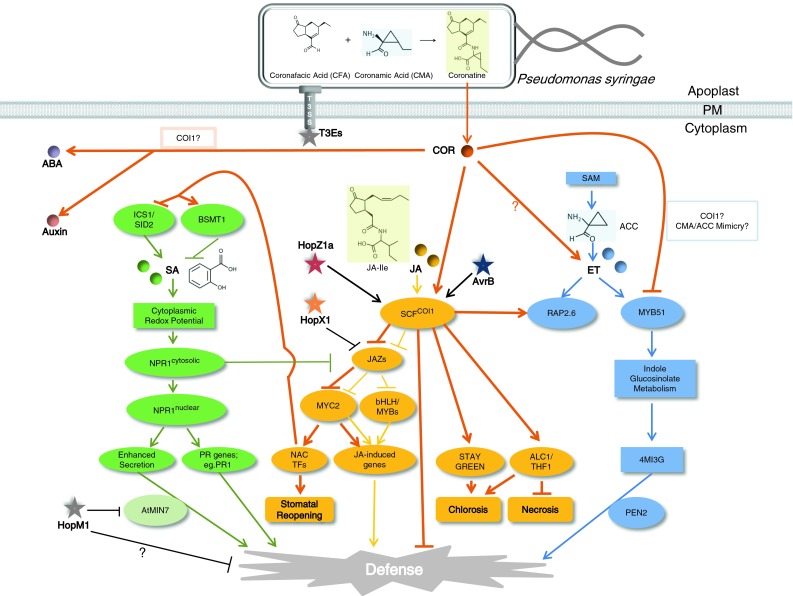
Roles of coronatine and type III effectors in modulating defense-related hormone signaling**. (**1) Roles of coronatine. Coronatine (COR) is composed of two moieties: coronafacic acid (CFA) and coronamic acid (CMA) (Bender et al. 1999). Once COR moves into the plant cell (presumably through diffusion), it activates JA-signaling through mimicking JA-amino acid conjugates such as (+)-7-JA-isoleucine (JA-Ile) shown in the model. COR is able to interact with SCF^COI1^ receptor complex with modestly higher affinity than JA-Ile (Sheard et al. 2010; Katsir et al. 2008; Fonseca et al. 2009b). Like JA-Ile, COR serves as ‘molecular glue’ between the receptor complex SCF^COI1^ and the negative regulator JAZ protein (Sheard et al. 2010), and triggers the degradation of JAZ through 26S proteosomal-mediated pathway (Chini et al. 2007; Thines et al. 2007). Upon JAZ degradation, positive regulator TFs (e.g. MYC2, bHLH, and MYBs) are released from suppression, and activate JA-responsive genes (Wasternack and Hause 2013). MYC2 also regulates several NAC TFs that suppress SA accumulation through regulating SA-biosynthesis gene ICS1 and SA modifying gene BSMT1. These NAC TFs were also found to be required for stomatal reopening induced by COR (Zheng et al. 2012). In return, SA-activated, cytosolic NPR1 monomers suppress the JA-signaling pathway. COR’s ability to contribute to chlorotic disease symptoms is also mediated through COI1 (Mecey et al. 2011). COR is able to suppress callose deposition through inhibiting an ET-dependent indole glucosinolate pathway where the role of COI1 is unknown (Geng et al. 2012; Millet et al. 2010). Perhaps the CMA moiety of COR mimics the ET precursor ACC, and interferes with ET production. Additionaly, COR perturbs auxin and ABA signaling which could potentially offset the restriction of bacterial growth caused by flg22-induced suppression of auxin signaling (Navarro et al. 2006) or ABA-induced stomatal closure (Melotto et al. 2006), respectively. Whether COI1 is engaged in auxin and/or ABA perturbation is unknown. 2) Roles of type III effectors. AvrB or COR, cooperatively with other T3Es and dependent on COI1, induce expression of an ET responsive factor—RAP2.6 (He et al. 2004). HopZ1a acetylates JAZ proteins, causing them to become destabilized dependent on COI1, and restores virulence to a cor- mutant of *Pto* DC3000 (Jiang et al. 2013). HopX1 directly destabilizes JAZ proteins without a requirement for COI1, likely via its cysteine protease activity, and restores virulence to a cor- mutant of *Pto* DC3000. HopX1 shares additional activities with COR, including reopening of stomata, causing plant cells to lose chlorophyll, and induction of chlorosis in susceptible plants (Gimenez-Ibanez et al. 2014). HopM1 affects SA-dependent secretory pathway through interacting with and degrading an ARF-GEF family protein involved in vesicle trafficking called AtMIN7 (Nomura et al. 2006). HopM1 is also functionally redundant with COR in suppressing an SA-independent defense sector of which the mechanism is unknown (Geng et al. 2012). *Solid lines* indicate established interactions. *Question marks* indicate unknown mechanisms. Hormone/coronatine/effector-specifc functions are color coded: *bold orange lines* coronatine-related functions, *yellow lines* JA-related functions, *green lines* SA-related functions, *blue lines* ET-related functions, *black lines* T3Es-related functions. Hormones are color coded, and indicated by *solid circles*. Type III effectors are color coded, and indicated by *solid stars*. Structural similarities between compounds are indicated by same color shading of the respective chemical structures

COR is composed of two moieties, the polyketide coronafacic acid (CFA) and coronamic acid (CMA) (Bender et al. 1999; Ichihara et al. 1977; Mitchell 1985; Parry et al. 1993). CMA is derived from L-alloisoleucine, a diastereomer of l-isoleucine activated by the nonribosomal peptide synthetase adenylation domain of CmaA (Rohde et al. 1998; Buell et al. 2003; Worley et al. 2013). CFA is synthesized from a cyclopentenone compound with subsequent modification carried out by genes of the *cfa* operon (Gross and Loper 2009). Coronafacate ligase, one of nine open reading frames within *cfa* operon, joins CFA and CMA with an amide linkage to form COR (Bender et al. 1993; Liyanage et al. 1995) (Fig. 1).

CFA and CMA are synthesized independently and the operons encoding the COR biosynthetic genes differ between *P. syringae* pv. tomato (*Pto*) strain DC3000 and *P. syringae* pv. *glycinea* (*Pgl*) strain 4180 (Worley et al. 2013; Sreedharan et al. 2006). The COR biosynthesis operons are encoded on a 90-kb plasmid in *Pgl* 4180 (Bender et al. 1993). On the other hand, the COR biosynthetic genes of *Pto* DC3000 exist within two distinct chromosomal clusters; the CFA operon is separated by ~26 kb of intervening DNA from the CMA biosynthesis genes and the adjacent genes regulating COR expression (Brooks et al. 2004). Biosynthesis of COR, as well as CFA and CMA, is thermo-regulated in *Pgl* 4180 and several other pathovars of *P. syringae* (Rohde et al. 1998). Consistent with the symptom development in infected plants, COR production is negligible at 30 °C and reaches maximal level at 18 °C (Bender 1999; Rohde et al. 1998). On the other hand, COR production is not thermo-regulated in *Pto* DC3000 and the production is much less in vitro (Braun et al. 2008; Weingart et al. 2004). This is due to the difference of a histidine protein kinase CorS between two strains (Braun et al. 2008; Smirnova et al. 2008; Weingart et al. 2004; Ullrich et al. 1995), although the specific mechanism is not yet clear.

COR both structurally and functionally mimics the most active isoleucine conjugate of JA (+)-7-iso-JA-Ile (JA-Ile) (Wasternack and Xie 2010; Fonseca et al. 2009b) (Fig. 1). The functional resemblance between COR and JA-Ile has been widely noted (Chini et al. 2007; Thines et al. 2007; Sheard et al. 2010; Glazebrook 2005; Gimenez-Ibanez and Solano 2013; Haider et al. 2000; Weiler et al. 1994) and is now demonstrated experimentally by solved crystal structures of each molecule in association with a COI1 (COR-insensitive 1) receptor complex (Sheard et al. 2010).

In addition to the proven ability of COR to mimic JA-Ile, similarity between the CMA moiety of COR and 1-aminocyclopropane-1-carboxylic acid (ACC) has been noted (Brooks et al. 2004) (Fig. 1). ACC, the rate-limiting precursor of ethylene (ET) biosynthesis in higher plants, and CMA each contain a cyclopropane ring. Although the individual moieties of COR (CMA and CFA) show very limited activity in plant tissues (Uppalapati et al. 2005), intact COR perturbs ET homeostasis or known outputs of ET-signaling (Kenyon and Turner 1992; Ferguson and Mitchell 1985; Geng et al. 2012; Millet et al. 2010) leading to the hypothesis that COR, through its CMA moiety, perturbs ET biosynthesis or signaling. However, since a direct effect of COR on ET-signaling, for example via mimicry of ACC by the CMA moiety, has not been demonstrated, the possibility that COR modulates ET-signaling indirectly cannot be ruled out.

## Suppression of plant defense and promotion of disease symptoms by COR

### COR activates JA-signaling by mimicking JA-Ile

COR makes multiple contributions to bacterial virulence, several of which are mediated via its ability to mimic bioactive jasmonates (Fig. 1). Jasmonates (JAs) are lipid-derived plant hormones that regulate a broad range of plant cellular and physiological responses to control plant growth and development, as well as responses to biotic and abiotic stresses (Wasternack and Hause 2013). The final step of converting JA to its active version is carried out by JAR1 (for this and other plant genes discussed, refer to Table 2). JAR1 is a jasmonate:amino acid synthetase that conjugates JA to several amino acids, notably creating bioactive JA-Ile (Staswick et al. 2002; Suza and Staswick 2008). Among biotic stress responses, JA-signaling typically is activated when plants are attacked by necrotrophic pathogens or herbivores (Hopke et al. 1994; Norman et al. 1999; Schenk et al. 2000; Stotz et al. 2000; Karban and Baldwin 1998; Pieterse et al. 2012).

**Table 2:** Genes discussed in this review

Gene	Function of gene product	References
*ABA3*	ABA biosynthesis	Leon-Kloosterziel et al. (1996)
*ALC1*	Mediates COR response in *N. benthamiana*	Wangdi et al. (2010)
*ASK1, ASK2*	Component of the SCF family of E3 ubiquitin ligases	Gray et al. (2001)
*AtCUL1*	Component of the SCF family of E3 ubiquitin ligases	del Pozo and Estelle (1999)
*AtMIN7*	ADP ribosylation factor-guanine nucleotide exchange factor	Nomura et al. (2006)
*COI1*	Receptor component of SCF^COI1^ complex	Xu et al. (2002)
*GRX480*	Glutaredoxin family regulator of redox state	Ndamukong et al. (2007)
*JAR1*	Conjugates jasmonic acid (JA) to amino acids	Staswick et al. (2002)
*MYB21*	R2R3-MYB transcription factor, JA-induced regulator of stamen development and defense	Cheng et al. (2009), Song et al. (2011)
*MYB51*	R2R3-MYB transcription factor, regulator of indole glucosinolate biosynthesis	Qi et al. (2011), Song et al. (2011)
*MYB75*	R2R3-MYB transcription factor, regulator of anthocyanin accumulation and trichome initiation	Qi et al. (2011)
*MYC2*	MYC-related transcriptional activator, central regulator of JA-signaling	Chini et al. (2007)
*NahG*	Bacterial salicylate hydroxylase, prevents accumulation of SA when expressed *in planta*	Delaney et al. (1994), Gaffney et al. (1993)
*NINJA*	Novel interactor of JAZ, function as negative regulators of jasmonate responses	Pauwels et al. (2010), Pauwels and Goossens (2011), Shyu et al. (2012)
*NPR1*	Redox-regulated transducer of SA signal, putative receptor for SA	Cao et al. (1994), Wu et al. (2012)
*NPR3, NPR4*	NPR1 homologs, putative receptors for SA	Fu et al. (2012)
*NYE1/SGR*	Regulator of chlorophyll degradation	Ren et al. (2007)
*ORA59*	AP2/ERF domain transcription factor, an essential integrator of the JA and ET-signaling pathways	Pré et al. (2008)
*OST1*	Guard cell specific kinase	Mustilli et al. (2002)
*PEN2*	Atypical myrosinase that hydrolyzes 4-methoxy indol-3ylmethylglucosinolate (4MI3G)	Lipka et al. (2005)
*PR* genes	Pathogenesis-related proteins, various functions	Uknes et al. (1992)
*RAP2.6*	Ethylene response factor subfamily B-4 transcription factor of ERF/AP2 family	He et al. (2004)
*SID2(EDS16)*	Isochorismate synthase, required for the majority of defense-associated SA production	Wildermuth et al. (2001)
*TGA2,TGA3,* *TGA5,TGA6,* *TGA7*	Transcription factors of the B-ZIP family that interact with NPR1 to regulate PR gene expression.	Zhou et al. (2000), Zhang et al. (1999), Kim et al. (2002), Despres et al. (2000)
*THF1*	ALC1 homolog in Arabidopsis	Wang et al. (2004)
*TPL*	Groucho/Tup1-type co-repressor TOPLESS (TPL), as general co-repressors that affect multiple signaling pathways including JA-signaling pathway	Szemenyei et al. (2008), Pauwels et al. (2010), Pauwels and Goossens (2011), Shyu et al. (2012)
*WRKY70*	WRKY-family transcription factor, activator of SA-induced genes, repressor of JA-induced genes.	Li et al. (2004)

Similar to signaling by other plant hormones such as auxin (Dharmasiri et al. 2005) and gibberellic acid (Yamaguchi 2008; Schwechheimer and Willige 2009), JA-signaling results from the proteasome-mediated removal of transcriptional repressors. In the case of JA-signaling, these repressors are called JAZ (containing Jasmonate ZIM domain) proteins (Chini et al. 2007; Thines et al. 2007). When levels of bioactive JA-Ile are low, JAZ proteins are stable and function as transcriptional repressors by physically interacting with a variety of transcription factors (TFs), including MYC2, a basic-helix loop helix TF that activates a significant proportion of JA-induced responses (Lorenzo et al. 2004; Dombrecht et al. 2007). Transcriptional repression by JAZ proteins occurs through recruitment of the general co-repressor TOPLESS (TPL), usually via the adapter protein Novel Interactor of JAZ (NINJA) (Pauwels et al. 2010; Pauwels and Goossens 2011; Shyu et al. 2012; Szemenyei et al. 2008).

The JAZ family in Arabidopsis has 12 members (Chini et al. 2007). In addition to MYC2, JAZ proteins also interact with two other MYC2-related bHLH TFs, which regulate overlapping as well as distinct responses with MYC2, and other bHLH/MYB transcriptional factors, like MYB75 and MYB21, which also regulate JA responses (Cheng et al. 2009; Qi et al. 2011; Song et al. 2011). Additionally, interactome and functional analyses indicate that JAZ proteins likely interact with a wide variety of TFs to regulate development and stress responses (Kazan and Manners 2012; Qi et al. 2011; Seo et al. 2011; Song et al. 2011). For example, Song et al. (2011) found that JAZ1, JAZ8 and JAZ11 interact with MYB21 and MYB24 in both yeast and planta to mediate JA-regulated development processes. Similarly, Seo et al. (2011) found that OsJAZ1 interact with OsbHLH148 to regulate JA-regulated drought stress in rice. Specificity of individual JAZ proteins for diverse transcription factors likely contributes to tuning the JA-response to specific contexts, such as in different cell-types, developmental stages, and stresses, via integration with other signaling pathways.

JA-signaling is activated when JAZ proteins are destabilized by proteasome-mediated degradation. A typical SCF ubiquitin ligase complex consists of an F-box protein in complex with SKP1 and a Cdc53 (Hershko and Ciechanover 1998; Deshaies 1999). In Arabidoposis, the F-box protein COI1 associates with Skp1-like proteins ASK1, ASK2 (Gray et al. 1999; Gray et al. 2001) and Cdc53-like protein AtCUL1 (del Pozo and Estelle 1999) to assemble the SCF^COI1^ E3 ubiquitin ligase complex (Devoto et al. 2002; Xu et al. 2002). JA-Ile binding to co-receptor complexes composed of a JAZ protein and COI1 within SCF^COI1^ triggers ubiquitination of the JAZ proteins. The resulting 26S proteasome-mediated degradation relieves JAZ-mediated transcriptional repression to activate JA-responsive gene expression (Fonseca et al. 2009a; Katsir et al. 2008). High affinity binding of COI1 complexes to JAZ proteins requires both JA-Ile (or COR) and inositol pentakisphosphate, which interacts with both COI1 and JAZ adjacent to the ligand (Sheard et al. 2010). A recent report indicates that COI1 has a function additional to functioning as a receptor for active JA-conjugates. The vascular pathogen, Verticillium longisporum, requires a COI1 activity that is independent of JA or JA-mimicry to complete its life cycle in Arabidopsis, indicating an unknown function of COI1 during the *V. longisporum*-plant interaction (Ralhan et al. 2012).

It has been long known that significant overlap exists between COR- and JA-signaling in tomato (Palmer and Bender 1995). Structural and pharmacological studies revealed that COR, as a structural mimic of JA-Ile, binds with high affinity to Arabidopsis COI1 (Sheard et al. 2010). The most active diastereomer for promoting pull down of plant expressed COI1 by JAZ proteins and for promoting anthocyanin accumulation in wild-type and *jar1* mutant Arabidopsis seedlings is (+)-7-iso-JA-Ile (Fonseca et al. 2009b). The cyclopentanone ring of COR is a stereoisomer of and demonstrates slightly higher activity than (+)-7-iso-JA-Ile in these assays (Wasternack and Xie 2010; Fonseca et al. 2009b). Also, COR may be resistant to catabolic and epimeric inactivation of JA-Ile (Fonseca et al. 2009b; Koo and Howe 2012). Thus, not only does COR mimic the active JA-Ile conjugate, but it may also function as a hyperactive agonist of JA-signaling.

COR and JA-Ile contact not only COI1, but also the JAZ protein within the COI1-JAZ co-receptor (Sheard et al. 2010), which raises the interesting possibility that COR is biased, relative to JA-Ile, toward specific COI1-JAZ co-receptors. For example, the JA-Ile interacting degron of Arabidopsis JAZ proteins is sequence divergent in JAZ7/8. While this variation renders JAZ8 largely insensitive to JA-Ile, COR retains, albeit at a lower affinity than for other JAZ proteins, the ability to bind and induce degradation of JAZ8 (Shyu et al. 2012). Differences in the ability of COR to target different JAZ proteins could be interpreted in two, non-mutually exclusive ways. Selective targeting of specific JAZs could reflect a “fine-tuning” of transcriptional activation by COR. Alternatively, the inability of COR to target individual JAZs, including alternative C-terminal splice variants with reduced affinity for JA-Ile and COR (Moreno et al. 2013; Chung et al. 2010), could reflect an adaptive, counter-defense strategy of the plant to resist the effect of COR (Chung et al. 2009). In either (or both) case(s), understanding the JAZ-selectivity of COR and JA-Ile is an important area to be explored.

### COR suppresses SA-signaling via antagonistic SA-JA crosstalk

Hormone crosstalk is used to fine-tune defense responses against biotic challengers with distinct lifestyles. To exploit these networks to their benefit, numerous plant pathogens produce hormones, hormone mimics, or effectors that stimulate plant production of hormones or modulate hormone signaling. An example of hormone crosstalk relevant to biotic defense occurs between the SA- and JA-dependent signaling pathways (Fig. 1). Generally, the JA/SA balance dictates whether plants mount defense responses tailored to necrotrophic pathogens and herbivores, by favoring JA-signaling, or to biotrophic and hemibiotrophic pathogens, by favoring SA-signaling (Baldwin et al. 1994; Creelman and Mullet 1997; Gimenez-Ibanez and Solano 2013; Kessler and Baldwin 2002; Paschold et al. 2008; Petersen et al. 2000; El Oirdi et al. 2011; Gao et al. 2011; Spoel et al. 2003).

Salicylic acid (SA) is a key phytohormone in plant defense against a variety of biotrophic and hemibiotrophic pathogens, including bacterial strains producing COR (Fig. 1). SA is a monohydroxybenzoic acid that mediates changes in redox potential, probably through S-nitrosylation and thioredoxin activity, when it accumulates in plant cells (Tada et al. 2008). A key protein in SA-signaling is NPR1 (Nonexpresser of PR genes 1) (Cao et al. 1997). The SA-induced redox change leads to the reduction of cytosolic, thiol-linked NPR1 oligomers to monomers that translocate to the nucleus (Cao et al. 1994; Kinkema et al. 2000; Mou et al. 2003). NPR1 monomers activate expression of pathogenesis responsive (*PR*) genes (Uknes et al. 1992) through interaction with TGA TFs, including TGA2, TGA3, TGA5, TGA6, and TGA7, that bind to activator sequence-1 (*as*-*1*) or *as*-*1*-like promoter elements (Fan and Dong 2002; Zhou et al. 2000; Zhang et al. 1999; Kim and Delaney 2002; Gimenez-Ibanez and Solano 2013; Despres et al. 2000). Through interaction with TL1-binding factor 1, an HSF-like transcription factor, nuclear-localized NPR1 also activates genes with TL1 promoter elements that support secretion of PR, and perhaps other classes of proteins, through the ER (Pajerowska-Mukhtar et al. 2012; Wang et al. 2005). Additionally, NPR1 induces expression of several WRKY TFs that function as both activators and suppressors of defense (Wang et al. 2006). During its activation by SA, NPR1-phosphorylation facilitates targeting of NPR1 by a Cullin3-based ubiquitin ligase and proteosome-mediated NPR1 turnover is required for full induction of NPR1 target genes (Spoel et al. 2009). One recent report indicated that NPR3 and NPR4, two paralogues of NPR1, are SA-receptors in Arabidopsis that function as adaptors to mediate NPR1 degradation (Fu et al. 2012). A second recent report used equilibrium dialysis ligand binding to show that NPR1 is itself an SA-receptor (Wu et al. 2012). Thus, clearly defining the nature of the SA-receptor(s) remains an important area for further work.

SA plays a central role in regulating plant biotic defenses. In addition to activating defense against biotrophs, for example through inducing expression of defense-promoting secretory genes and antimicrobial *PR* genes, SA-signaling also has an antagonistic effect on JA-signaling (Fig. 1). Induction of JA-responsive genes is suppressed by SA when SA and MeJA are together exogenously applied to Arabidopsis plants. In SA-deficient *NahG* plants infected by *P. syringae*, JA accumulates to 25-fold higher levels and consequently JA-responsive genes are expressed to higher levels (Spoel et al. 2003; Glazebrook et al. 2003). Activation of SA-signaling by *P. syringae* suppresses JA-signaling and thus renders plants more susceptible to a necrotrophic pathogen (Spoel et al. 2007). NPR1 plays a crucial role in SA-mediated inhibition of JA-dependent signaling, with mechanisms including induced expression of the glutaredoxin GRX480 and the WRKY70 transcription factor as well as destabilization of the ORA59 (OCTADECANOID-RESPONSIVE ARABIDOPSIS AP2/ERF domain protein 59) (Li et al. 2004; Ndamukong et al. 2007; Van der Does et al. 2013; Pre et al. 2008). Also, type II TGA factors are essential for the ability of SA to suppress the ET-signaling contribution to the expression of Arabidopsis genes induced dependent on both JA- and ET-signaling (Zander et al. 2014). Notably, cytosolic, but not nuclear, NPR1 is required for crosstalk, indicating that NPR1 has distinct roles in SA-signaling and suppression of JA-signaling (Spoel et al. 2003). The significance of the suppressive effect of SA on JA-signaling is supported by the observation that it occurs in numerous Arabidopsis accessions treated with SA, MeJA, or both in combination (Koornneef et al. 2008).

The inhibitory crosstalk of SA-signaling toward JA-signaling is mirrored by JA-mediated suppression of SA-signaling (Fig. 1). To exploit this crosstalk, *P. syringae* produces COR to hijack JA-signaling and suppress SA-mediated defense. Compared to wild-type plants, *Pto* DC3000 infection of *coi1*-*20* plants elicits elevated levels of SA and *PR* gene expression, and bacterial growth is suppressed. Bacterial multiplication is recovered in *coi1*-*20* plants expressing *NahG* (Kloek et al. 2001). Also, cor- (a COR-deficient mutant of *Pto* DC3000) bacteria induced less JA- and more SA-responsive gene expression, and the reduced growth of cor- strains was restored when the bacteria infected SA-signaling deficient plants (Brooks et al. 2005; Uppalapati et al. 2007; Geng et al. 2012). Thus, studies utilizing both plant and bacterial mutants indicate that COR stimulates JA-signaling to suppress SA-signaling and that SA-signaling is a necessary component of the defense suppressed by COR. A mechanism for suppression of SA-signaling by COR is through *MYC2*-dependent expression of three NAC TFs that both 1) repress expression of genes controlling SA-biosynthesis, including an isochorismate synthase (SID2), and 2) induce expression of a benzoic/salicylic acid carboxyl methyltransferase (BSMT1) that reduces the pool of free (biologically active) SA via methylation (Zheng et al. 2012).

The ability of COR to inhibit defense signaling in plant cells extends beyond its ability to promote JA-signaling. In addition to causing analogous plant physiological responses as JA, such as inhibiting root elongation, inducing anthocyanin production, and promoting senescence, COR also causes responses not associated with JA, including cell wall thickening and changes to chloroplast structure in tomato plants (Palmer and Bender 1995). Furthermore, exogenous treatment with COR causes hypertrophy and increased amylase activity in potato tuber tissue and also causes anomalous cell growth in tobacco leaves (Kenyon and Turner 1992; Sakai et al. 1979; Sakai 1980; Bender et al. 1999; Feys et al. 1994). More recently, COR was shown to inhibit defensive fortification of cell walls independent of targeting COI1 and suppressing SA-signaling (Geng et al. 2012). Thus, in addition to its well-documented ability to dampen SA-signaling, at least some of the virulence activity of COR is independent of the inhibitory crosstalk between JA- and SA-signaling pathways, and even independent of its ability to target COI1.

### COR induces stomatal reopening

A critical first step in the disease cycle of epiphytic phytopathogenic bacteria is the ability to enter the intracellular spaces of plant tissues. Stomata are natural openings and a key portal exploited for bacterial invasion. Guard cells not only regulate gas exchange and water transpiration, but stomatal closure is also an important strategy for plants to prevent the ingress of pathogens, such as *P. syringae*. Melotto et al. (2006) discovered that *P. syrinage* on leaf surfaces congregate at stomata and that stomatal closure triggered by recognition of Pathogen Associated Molecular Pattern (PAMPs) is a plant counter-defense strategy to prevent bacterial entry (Melotto et al. 2006). At least two hormone signaling pathways, SA and abscisic acid (ABA), are critical to PAMP-triggered stomatal closure. Stomatal closure was not observed in SA-deficient *nahG* transgenic plants and SA-biosynthetic mutant *sid2* (also known as *eds16*) plants (Wildermuth et al. 2001). Extensive studies have shown that ABA is required for stomatal closure when plants are under abiotic stress (Cummins et al. 1971; Fan et al. 2004; Mustilli et al. 2002; Tardieu and Davies 1992). PAMP-induced stomatal closure also was not observed in *ost1* kinase mutant and ABA-deficient *aba3* mutant plants (Leon-Kloosterziel et al. 1996; Melotto et al. 2006; Mustilli et al. 2002).

COR exploits a role for JA-signaling in PAMP-induced stomatal closure. Similar to treatment with purified PAMPs, *Pto* DC3000 causes stomata to close. But unlike PAMPs, the bacteria quickly reverse the closure, thus allowing for bacterial invasion into the apoplast. The ability of *Pto* DC3000 to overcome the PAMP-induced stomatal defense is dependent on COR; a cor- strain fails to reopen closed stomata. Furthermore, COR inhibits ABA-induced stomatal closure in a *COI1*-dependent manner (Melotto et al. 2006). Thus, COR is critical for the ability of *P. syringae* to overcome PAMP-induced stomatal closure by a mechanism that acts either on or downstream of both SA- and ABA-dependent processes. The same NAC TFs through which COR suppresses SA accumulation also contribute to the ability of COR to overcome ABA-induced stomatal closure and to reopen stomata during *P. syringae* infection (Zheng et al. 2012).

### COR promotes chlorotic disease symptoms in infected plants

Mutant strains of *P. syringae* unable to produce COR elicit reduced disease symptoms including little or no chlorosis (Feys et al. 1994; Bender et al. 1987; Bender et al. 1999; Kloek et al. 2001; Brooks et al. 2004; Brooks et al. 2005; Block et al. 2005; Mittal and Davis 1995). Treatment of tomato leaves with exogenous COR induced shrunken and descended chloroplasts located near the bottom of the palisade mesophyll cells (Uppalapati et al. 2005). Possibly related to this observation, COR or MeJA repress expression of a large number of genes involved in chloroplast metabolism, including genes encoding chlorophyll a/b binding proteins and thylakoid luminal proteins (Palmer and Bender 1995; Uppalapati et al. 2005; Attaran et al. 2014). Despite these long-standing observations, the molecular basis of how COR contributes to chlorosis is just beginning to be understood.

In Arabidopsis, COR alone induces anthocyanin accumulation (Bent et al. 1992; Feys et al. 1994). However, in the context of an infection by *Pto* DC3000, COR contributes to chlorotic disease symtpoms. Screening for Arabidopsis mutant plants that do not display chlorosis after infection by *Pto* DC3000 identified a “no chlorosis” mutant (Mecey et al. 2011). Unlike in wild-type plants, the chlorophyll levels in the mutant are relatively unchanged after infection. The mutation causes an amino acid substitution in the nuclear-encoded, chloroplast-localized Staygreen/Non-Yellowing/Mendel’s I locus (SGR) protein. SGR is associated with chlorophyll degradation. Mutation of *SGR* causes a stable, non-yellowing phenotype during senescence of leaves (Ren et al. 2007). Exogenously applied COR and *Pto* DC3000 induce *SGR* expression in a COI1-dependent manner and, conversely, cor- bacteria induce low levels of SGR compared to the wild-type bacteria. Thus, activating expression of SGR by targeting of COI1 (and thus likely by mimicking JA-Ile) plays a critical role in the contribution of COR to the induction of chlorotic disease symptoms by *Pto* DC3000 (Mecey et al. 2011).

Wangdi et al. 2010 used virus-induced gene silencing to identify several genes with altered COR (ALC) responses following exogenous application of COR. Silencing of *ALC1* in *N. benthamiana* and tomato resulted in a COR-induced necrotic phenotype that occurs without visible chlorosis. In addition to the lack of COR-induced chlorotic symptoms, *Pto* DC3000 infection of tomato with silenced *ALC1* or Arabidopsis with a mutation of the *ALC1* homolog (*THF1*) induced accelerated, coalescing necrotic lesions without apparent chlorosis (Wangdi et al. 2010). ALC1/THF1 is localized in the chloroplast and ALC1 is destabilized by COR in *N. benthamiana* leaves (Wangdi et al. 2010; Wang et al. 2004). Since the ability of COR to destabilize ALC1 and to cause necrotic lesions without chlorosis depends on COI1, this activity likely results from its mimicry of JA (Wangdi et al. 2010). Thus, *ALC1* links activation of COI1 by COR to both chlorotic and necrotic disease symptoms. SGR-mediated chlorophyll breakdown promotes the production of defense-promoting reactive oxygen (Mur et al. 2010) and ALC1/THF1 is speculated to play a role in maintenance of reactive oxygen homeostasis (Wangdi et al. 2010). Thus, the effects of COR highlight the importance of chloroplast physiology, including chloroplast-derived reactive oxygen, during *P. syringae* infection.

### COR disrupts defense-associated secondary metabolism and cell wall reinforcement

Secondary metabolites are not necessary for plant growth and development in pristine growth conditions, but provide important and sometimes essential functions when plants growing in natural conditions are subjected to biotic or abiotic stresses. JA-signaling plays a crucial role in regulating plant secondary metabolites in both a COI1-dependent and a COI1-independent manner (Devoto et al. 2005). Similarly, COR regulates primary and secondary metabolism during *P. syringae* infection of Arabidopsis, including the induction of genes involved in tryptophan synthesis, anthocyanin synthesis, and methionine-derived glucosinolates (Thilmony et al. 2006). Treatment of Arabidopsis with purified COR also induces the expression of genes involved in glucosinolate and phenylpropanoid metabolism (Attaran et al. 2014). Indolic compounds constitute one branch of the phenylpropanoid pathway. One fate of indole rings is as intermediates in the synthesis of tryptophan that in turn can serve as a precursor to secondary metabolites involved in plant defense, such as benzoxazinoids, indole glucosinolates (IGs) and the phytoalexin camalexin (Ahmad et al. 2011; Bednarek et al. 2009; Frey et al. 1997). Of these myriad potential effects of COR, perturbation of IGs metabolism has of late come into focus as a potentially critical means by which COR suppresses host defense.

IGs are a class of thioglucosides that have been well documented to play a role in the resistance to chewing insects (Bednarek et al. 2009; Clay et al. 2009; Halkier and Gershenzon 2006; Kim and Jander 2007). More recent studies have demonstrated that tryptophan-derived IGs also play a significant role in defense responses of living tissue against microbes (Bednarek et al. 2009; Clay et al. 2009). Callose, a glucan polymer, is deposited as part of cell wall appositions, which are physical barriers formed at pathogen infection sites. The deposition of callose induced by the PAMP flg22 is well studied in Arabidopsis (Clay et al. 2009; Kim and Mackey 2008). Both ET-signaling and IGs are required for PAMP-induced callose deposition in the leaves of liquid-grown Arabidopsis seedlings (Clay et al. 2009). MYB51, a TF involved in the regulation of IGs biosynthesis, is induced dependent on ET-signaling and is required for the response. A role for IGs in PAMP-induced callose deposition was demonstrated by the lack of callose in mutant seedlings deficient in IGs biosynthesis. 4-methoxyindol-3-ylmethylglucosinolate (4MI3G), an IG candidate found by metabolic profiling, rescued callose deposition in seedlings unable to produce 4MI3G. Furthermore, an unknown hydrolysis product(s) from degradation of 4MI3G by PEN2, an atypical myrosinase (Bednarek et al. 2009; Lipka et al. 2005), are also required for PAMP-induced callose deposition (Clay et al. 2009). Similarly, IGs metabolism and 4MI3G are important for broad-spectrum, penetration-stage resistance of plants to biotrophic fungal pathogens (Bednarek et al. 2009), perhaps also through regulation of cell wall-associated defense. It was recently shown that COR suppresses IGs metabolism and PEN2-dependent callose deposition during *P. syringae* infection of Arabidopsis (Geng et al. 2012).

It is an interesting paradox that COR inhibits the production of specific IGs, including 4MI3G and presumably downstream products necessary for callose deposition, while it more generally stimulates the expression of genes that promote the production of IGs. The mechanism by which COR perturbs the production of specific IGs is unknown, but may be through perturbation of additional plant hormone signaling pathways. A first possibility is that COR generally perturbs metabolism of indole-containing compounds. Reduced production of IGs and increased expression of genes involved in tryptophan metabolism could relate to changes in the synthesis of the phytohormone auxin. Uppalapati et al. 2005 demonstrated that exogenous application of COR induces auxin-related gene expression in tomato, indicating that COR might promote bacterial virulence by perturbing auxin signaling (Uppalapati et al. 2005; Robert-Seilaniantz et al. 2011; Thilmony et al. 2006; Kazan and Manners 2009). In addition to offsetting the flg22-induced suppression of auxin signaling (Navarro et al. 2006), another consequence of COR promoting auxin production may be to limit indole availability for IGs production.

A second possibility is that COR affects IGs metabolism through perturbation of ET-signaling, which plays a key role in IGs metabolism (Fig. 1). COR causes accumulation of ACC, increased ACC-synthase (ACS) activity, and increased ET production in Bean and *Nicotiana tabacum* plant leaves (Ferguson and Mitchell 1985; Kenyon and Turner 1992). Whether COR directly or indirectly stimulates ET production is unclear, but considering the CMA moiety of COR is a structural mimic of ACC (Brooks et al. 2004); the effect of COR on production of ET from methionine might be direct. The stimulation of ET production by COR is counter-intuitive relative to its ability to suppress ET-dependent responses, i.e., IGs metabolism and callose deposition. However, this apparent contradiction may result from COR-mediated inhibition of ET production disrupting feedback regulation and thus ultimately leading to mis-timed and/or mis-regulated ET production. Hypotheses for how COR might initially inhibit ET production include interaction with ACS enzymes as a non-released substrate analog or with ACC oxidase enzymes as a competitive inhibitor of ACC.

The role of ET-signaling in IGs metabolism and callose deposition differs between Arabidopsis tissues and growth conditions. Both ET-signaling and IGs are required for PAMP-induced callose deposition in the roots of liquid-grown Arabidopsis seedling. Further experiments with seedling roots indicated that both ET-dependent and ET-independent mechanisms contribute to PAMP-induced expression of *MYB51* and that COR, dependent on COI1, inhibits callose deposition and suppresses *MYB51* expression in both an ET-signaling dependent and an ET-signaling independent manner (Millet et al. 2010). In the leaves of liquid-grown Arabidopsis seedlings, *MYB51* expression, IGs synthesis, and callose deposition were each dependent on ET-signaling (Clay et al. 2009). In the leaves of soil-grown plants, COR promoted bacterial multiplication and inhibited IGs metabolism and callose deposition (Geng et al. 2012). Surprisingly, COR was able to suppress callose deposition in the leaves of *coi1*-*16* mutant plants, indicating a COI1-indpendent defense suppressing activity of COR. Collectively, these studies indicate the existence of complicated mechanisms of PAMP-induced callose deposition in different plant tissues and growth conditions and point to the potential for multiple activities of COR suppressing these pathways.

It is unclear if the COI1-independent, defense suppressing activities of COR in Arabidopsis are mediated through mimicry of JA-Ile, ET, both or neither. Consistent with the JA-mimicry hypothesis, it has been shown that MeJA induces some genes independent of targeting COI1 (Devoto et al. 2005). COR, through its ability to mimic active JA-conjugates, could similarly alter gene expression independent of targeting COI1. One of the COI1-independent, MeJA-induced genes is an ACC-synthase (Devoto et al. 2005), indicating a possible mechanism for how COR might indirectly affect ET-signaling. An alternative and non-exclusive hypothesis is that the COI1-independent function of COR is also independent of its ability to mimic active JA-conjugates. An intriguing possibility is that the CMA moiety of COR, through mimicry of ACC, directly perturbs ET biosynthesis.

## COR and type III effectors cooperate to promote bacterial virulence

Bacterial pathogens deploy a variety of virulence factors, including toxins and type III effectors (T3Es), that work in a ‘multifunctional, cooperative, and redundant’ manner (Dean and Kenny 2009). As a result, bacteria often maintain their overall disease-causing ability even when one of their virulence strategies fails due to mutation or incompatibility on a given host. Consistent with this idea, several recent studies have indicated that the multifunctional COR toxin has functions that overlap with various T3Es in a cooperative or (semi-)redundant manner.

### Transcription remodeling by COR and type III effectors

An examination of Arabidopsis gene expression following infiltration with wild-type *Pto* DC3000, *Pto*cor-, or *Pto*cor- *hrpS* (a COR- and type III secretion system (TTSS)-deficient double mutant of *Pto* DC3000) revealed overlapping yet still distinct roles of COR and T3Es in reprogramming of the host transcriptome (Thilmony et al. 2006). Both COR and T3Es contribute to the regulation of genes responsive to auxin, ABA, and cytokinin, suggesting that *Pto* DC3000 utilizes multiple virulence factors to ensure the successful perturbation of the host hormone network. One unique effect of COR, the significant induction of JA- responsive genes, is in accordance with JA-mimicry by COR and earlier work in tomato showing COR from *Pto* DC3000 induces JA-related gene expression and contributes to virulence (Zhao et al. 2003). Another unique effect of COR likely related to JA-mimicry by COR, is the prominent modulation on genes involved in secondary metabolism consistent with induced expression of Arabidopsis genes involved in secondary metabolism by JAs (Sasaki-Sekimoto et al. 2005; Taki et al. 2005). Interestingly, some of the secondary metabolism-related genes are antagonistically regulated by COR and type III effectors, presumably due to the distinct functions of these virulence factors. Contrary to COR, the prominent function of T3Es was differential regulation of SA-related genes and suppression of basal defense-related genes. Suppression of a few basal defense-related genes by COR was also observed. This study defined effects of COR by comparing wild-type *Pto* DC3000 to the *Pto*cor- mutant, both of which deliver the full complement of T3Es. Thus, some effects of COR were likely masked by T3Es and more functional overlap is expected between COR and T3Es than revealed by this study alone. Examples of functional overlap between COR and specific T3Es are described in the following sections.

### Perturbation of hormone signaling by COR and T3Es

A variety of T3Es have been demonstrated to perturb hormone signaling pathways also targeted by COR. The T3Es AvrPto and AvrPtoB from *Pto* DC3000 induce ET production and signaling contributing to cell death in susceptible tomato plants (Cohn and Martin 2005). The ability of these T3Es to stimulate ET production correlates with their ability to induce the expression of two tomato ACC oxidase genes. Thus, in tomato, the virulence activity AvrPto and AvrPtoB might functionally overlap with that of COR. While functional redundancy between COR, AvrPto and AvrPtoB is speculative, the following paragraphs describe examples of overlapping function of COR with three different T3Es that target the JA-signaling pathway. Interestingly, relative to direct targeting of COI1 by COR, these T3Es perturb JA-signaling upstream of or at the COI1-signaling node.

The first example is AvrB, a T3E that targets upstream of COI1 (Fig. 1). He et al. 2004 showed that *Pto* DC3000, dependent on both COR and T3Es, induces the expression of an Arabidopsis ERF (ethylene responsive factor) gene, *RAP2.6*. Both a TTSS mutant and a cor- mutant of DC3000 failed to induce *RAP2.6*. Interestingly, AvrB complemented the ability of the cor- mutant strain to induce *RAP2.6*, indicating overlapping activities for the T3E and toxin. Further support for this overlap came from the observation that the ability of AvrB to induce *RAP2.6* was dependent on COI1 (He et al. 2004). Later work indicated the ability of AvrB to activate JA-signaling is mediated by targeting of MAP kinase 4 (Cui et al. 2010). Thus, AvrB appears to induce JA-response genes by activating JA-signaling upstream of COI1.

The other two effectors activate JA-signaling by targeting JAZ proteins (Fig. 1). HopZ1a from *P. syringae* pv. *syringae* strain A2 acetylates JAZ proteins causing them to become destabilized dependent on COI1 (Jiang et al. 2013). HopX1 from *P. syringae* pv. *tabaci* (*Pta*) strain 11528 destabilizes JAZ proteins without a requirement for COI1, likely via its cysteine protease activity that directly cleaves the central Zim domain of the JAZ proteins (Gimenez-Ibanez et al. 2014). Like COR, both HopX1 and HopZ1a can induce expression of JA-response genes, suppress SA-signaling, and restore virulence to cor- mutant *Pto* DC3000 (Jiang et al. 2013; Gimenez-Ibanez et al. 2014). HopX1 shares additional activities with COR, including reopening of stomata, causing plant cells to lose chlorophyll, and induction of chlorosis in susceptible plants (Gimenez-Ibanez et al. 2014). Since *Pta* 11528 does not produce COR, HopX1 may serve as an alternative evolutionary strategy to compensate for the lack of COR. It is interesting to consider whether HopZ1a and HopX1 will target all or a specific subset of JAZ proteins in host plants. When tested against a nearly complete set of Arabidopsis JAZ proteins, HopX1 targeted all and HopZ1a targeted a subset. One model is that JAZ-selectivity reflects fine-tuning of the virulence activity of a T3E. Another possibility is that resistant JAZ proteins, such as those derived from alternate splice variants or sequence divergent homologs, are present in co-evolved hosts to help overcome the effects of T3Es. In either case, HopX1, which comes from a *tabaci* pathovar of *P. syringae*, might be expected to target only a subset of JAZ proteins from tobacco plants.

### Suppression of cell wall defense by COR and T3Es

HopM1 is a T3E encoded by a gene located in the conserved effector locus (CEL) of *Pto* DC3000. HopM1 overcomes SA-dependent host immunity by destabilizing AtMIN7 to disrupt G-protein-mediated endomembrane trafficking as well as perturbing another Arabidopsis target(s) to disrupt an SA-independent pathway supporting Arabidopsis defense (Nomura et al. 2006; Gangadharan et al. 2013; Nomura et al. 2011) (Fig. 1). CEL, which is physically adjacent to the locus containing genes that encode the structural proteins of the type III secretion system apparatus, contains effectors important for the virulence of a variety of bacterial pathogens (Alfano et al. 2000; Badel et al. 2006; DebRoy et al. 2004; Ham et al. 2006; Kvitko et al. 2009). A recent study revealed a functional overlap between COR and HopM1 in suppressing cell wall-associated defense (Geng et al. 2012).

The ability of COR to suppress cell wall-associated defense escaped detection until recently (Geng et al. 2012; Millet et al. 2010) because the effect of COR is masked by T3Es of the CEL. Further obscuring this activity of COR, *Pto*ΔCEL (CEL deletion of *Pto* DC3000) elicits SA-signaling that overcomes the suppressive effect of COR. The new activity of COR was detected by examining defense responses against a *Pto*ΔCEL cor- (CEL deletion and COR-deficient double mutant strain) in SA-signaling deficient mutant plants (*sid2* and *npr1*). The *Pto*ΔCEL cor- double mutant elicited more callose and grew less than the *Pto*ΔCEL single mutant in SA-signaling mutant plants. Furthermore, those higher levels of callose elicited by *Pto*ΔCEL cor- were suppressed by either exogenous application of COR or expression of *hopM1* in the double mutant strain. Thus, COR and HopM1 carry out overlapping roles in suppressing cell wall-associated defense.

Although COR and T3Es of the CEL locus share the ability to suppress cell wall-associated defense, their mode of action differs. In SA-signaling competent plants, HopM1 suppressed the high levels of callose deposition induced by *Pto*ΔCEL cor- while COR could not. Thus, COR and HopM1 distinctly suppress signaling within the plant defense network by functioning in mechanistically distinct manners. The ability of COR to suppress callose deposition and promote bacterial growth in SA-signaling deficient mutants indicates that COR targets an SA-independent sector. Consistent with this idea, Geng et al. 2012 showed that COR perturbs IGs metabolism. Unlike COR that can only do so in SA-signaling deficient mutant plants, HopM1 suppresses callose deposition induced by *Pto*ΔCEL cor- and restores bacterial growth in both wild-type and SA-signaling deficient mutant plants. Thus, HopM1 suppresses both SA-dependent and SA-independent sectors, perhaps through downstream effects on defense-associated vesicle trafficking. The mode of action by which COR and HopM1 each target distinct sectors of a converged portion of the plant defense network remains to be elucidated.

## Conclusions and future questions

Plants consistently face environmental stresses, including biotic stresses, due to their sessile lifestyle. The key roles of hormone signaling and secondary metabolic pathways in the ability of plants to deal with these stresses make targeting of them an effective strategy deployed by plant bacterial pathogens to suppress host defense. Here, we reviewed the demonstrated ability of the phytotoxin COR to hijack JA-signaling and thus suppress SA-signaling. We also speculated about targeting of ET-signaling by COR and considered how one, or perhaps both, of these activities contributes to the various virulence activities of COR. The finding that COR promotes virulence independent of targeting COI1 opens a path to study this novel activity of COR separate from the confounding effects of COI1 activation. The perturbation of ET-dependent IGs metabolism provides an attractive system for this effort. In addition to producing hormones and/or hormone mimics, such as COR, bacteria also produce T3Es. COR cooperates with other T3Es to promote bacterial virulence and dampen the induced defense responses. The collaborative targeting of plant hosts by pathogen-produced virulence factors, for example by COR and T3Es that target plant hormone signaling pathways, is an area that, with further exploration, will reveal a better view of the elegant network comprising the plant immune system and how it is collaboratively defeated by pathogen-derived virulence factors.
